# A radiological cadaveric study of obturator nerve involvement and cranial injectate spread after different approaches to the fascia iliaca compartment block

**DOI:** 10.1038/s41598-023-39041-5

**Published:** 2023-07-26

**Authors:** Werner ten Hoope, Pascal S. H. Smulders, Holger M. Baumann, Jeroen Hermanides, Ludo F. M. Beenen, Roelof-Jan Oostra, Peter Marhofer, Philipp Lirk, Markus W. Hollmann

**Affiliations:** 1 Department of Anesthesiology, Amsterdam UMC Location University of Amsterdam, Meibergdreef 9, 1105 AZ Amsterdam, The Netherlands; 2 Department of Anesthesiology, Rijnstate Hospital, Wagnerlaan 55, 6815 AD Arnhem, The Netherlands; 3 Department of Radiology and Nuclear Medicine, Amsterdam UMC Location University of Amsterdam, Meibergdreef 9, 1105 AZ Amsterdam, The Netherlands; 4 Department of Medical Biology, Amsterdam UMC Location University of Amsterdam, Meibergdreef 9, 1105 AZ Amsterdam, The Netherlands; 5 Department of Anesthesia, Intensive Care Medicine and Pain Medicine, Medical University of Vienna, Spitalgasse 23, 1090 Vienna, Austria; 6 Department of Anesthesiology, Brigham and Women’s Hospital, Harvard Medical School, 75 Francis Street, Boston, MA 02115 USA

**Keywords:** Anatomy, Pain management, Surgery, Translational research, Pain

## Abstract

Whether the fascia iliaca compartment block (FICB) involves the obturator nerve (ON) remains controversial. Involvement may require that the injectate spreads deep in the cranial direction, and might thus depend on the site of injection. Therefore, the effect of suprainguinal needle insertion with five centimeters of hydrodissection-mediated needle advancement (S-FICB-H) on ON involvement and cranial injectate spread was studied in this radiological cadaveric study. Results were compared with suprainguinal FICB without additional hydrodissection-mediated needle advancement (S-FICB), infrainguinal FICB (I-FICB), and femoral nerve block (FNB). Seventeen human cadavers were randomized to receive ultrasound-guided nerve block with a 40 mL solution of local anesthetic and contrast medium, on both sides. Injectate spread was objectified using computed tomography. The femoral and lateral femoral cutaneous nerves were consistently covered when S-FICB-H, S-FICB or FNB was applied, while the ON was involved in only one of the 34 nerve blocks. I-FICB failed to provide the same consistency of nerve involvement as S-FICB-H, S-FICB or FNB. Injectate reached most cranial in specimens treated with S-FICB-H. Our results demonstrate that even the technique with the most extensive cranial spread (S-FICB-H) does not lead to ON involvement and as such, the ON seems unrelated to FICB. Separate ON block should be considered when clinically indicated.

## Introduction

Regional anesthesia (RA) techniques, such as fascial plane blocks and peripheral nerve blocks, have become a central component of multimodal analgesic strategies^1^. Benefits, in theory, include superior pain control, improved patient satisfaction, prevention of postoperative delirium, and reduced opioid consumption^2–4^. The use of RA is particularly relevant for (lower) extremity surgery due to its painful characteristics^5^. Numerous injection techniques targeting the hip, anterior thigh and knee have been developed, one of which is the fascia iliaca compartment block (FICB).

The origins of FICB can be traced back to Winnie’s 3-in-1 block, an anterior landmark approach to the lumbar plexus that targets the femoral nerve (FN), lateral femoral cutaneous nerve (LFCN) and obturator nerve (ON)^6^. Reports of inconsistent LFCN and ON blockade then led to studies that investigated the anatomical relations of the inguinal region, and local anesthetic distribution during 3-in-1 block^7,8^. These studies ultimately resulted in the establishment of a loss-of resistance technique, now known as FICB, targeting the fascia iliaca compartment (FIC)^8^. Later modifications of the technique include the incorporation of ultrasound guidance and more recently, the growing popularity of suprainguinal (S-FICB) over infrainguinal (I-FICB) injection sites^9–11^. However, although S-FICB was developed to acquire more consistent results when compared to I-FICB, debate persists over clinical superiority regarding coverage of the ON^12–20^.

**Figure 1:**
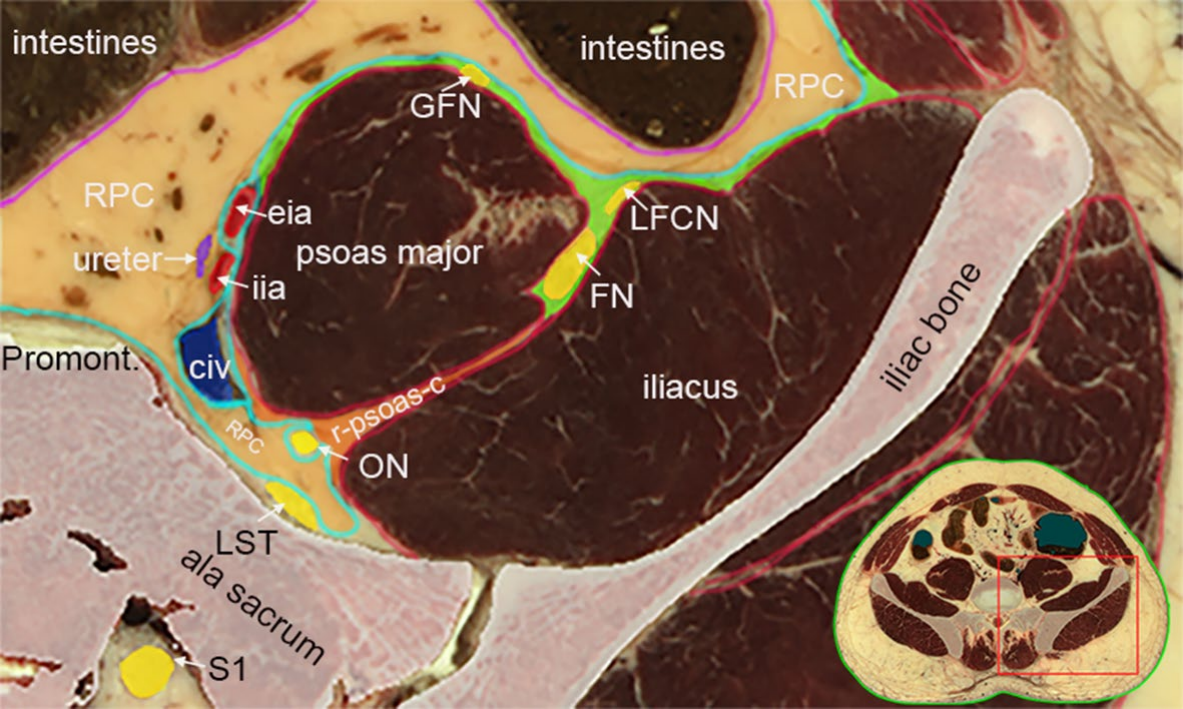
A cross-sectional image, cut at the level of the anterior superior iliac spine, explaining the anatomical location of the obturator nerve (ON). The ON arises from the anterior divisions of the second, third and fourth lumbar spinal nerves in the psoas compartment. It then enters the retro-psoas compartment at the posteromedial border of the psoas major muscle, posterior to the iliac arteries and vein. Here, it is separated from the FIC (shown in green) by the psoas major and iliacus muscles, and the fascia transversalis (TF; shown in cyan). The parietal peritoneum is shown in purple. Subsequently, the ON enters the retroperitoneal compartment by invaginating the TF, and runs down the pelvis until it exits through the obturator canal. Thus, the FIC never encompasses the ON, but whether it has a cranial opening that offers a route to the retroperitoneal compartment and ON remains debated. The figure is reprinted with permission from Thomas Fichtner Bendtsen^13^. *RPC* retroperitoneal compartment, *GFN* genitofemoral nerve, *LFCN* lateral femoral cutaneous nerve, *FN* femoral nerve, *eia* external iliac artery, *iia* internal iliac artery, *civ* common iliac vein, *ON* obturator nerve, *r-psoas-c* retro-psoas compartment, *LST* lumbosacral trunk.

Similar to other fascial plane blocks, FICB provides analgesia on the assumption that an injectate can open the potential space between the fascia iliaca (FI) anteriorly and the psoas major and iliacus muscles posteriorly, and spread towards the nerves within by means of bulk flow and diffusion^21^. However, recent anatomical reports have shown that the ON does not transverse the FIC, but rather passes through the psoas, retro-psoas and retroperitoneal compartments (Fig. 1)^12,13^. Consequently, local anesthetic (LA) is required to spread outside of the FIC in order to involve this nerve. While these studies agree on the closure of the FIC on its posteromedial border, they offer conflicting views on the existence of a cranially located opening to the retroperitoneal compartment and ON^12,13^. If this cranial path indeed exists, successful blockade of the ON with FICB would likely mandate overflowing the FIC, or a cranial injection site within the FIC.

Therefore, we set up a radiological cadaveric study to determine the effect of a cranial injection deep under the FI using suprainguinal needle insertion with five centimeters of additional hydrodissection-mediated needle advancement (S-FICB-H) on coverage of the ON. Further, we measured cranial injectate spread and involvement of the FN and LFCN. Results were compared with S-FICB without additional hydrodissection-mediated needle advancement, I-FICB, and femoral nerve block (FNB). We hypothesized that the injectate would reach most cranial for S-FICB-H, but that none of the nerve block techniques would result in reliable coverage of the ON.

## Methods

A convenience sample of 17 fresh, unfrozen and unembalmed adult human cadavers was included in this study. Cadavers were bequeathed to the Amsterdam University Medical Centers (location AMC) to serve research and educational purposes. Written informed consent for donation was given during life, in accordance with the Dutch Burial and Cremation act (BWBR0005009). The Medical Ethical Committee of the Amsterdam University Medical Centers (location AMC) provided a waiver for approval of this study. Further, experiments were performed according to relevant international and institutional regulations and ethics guidelines. The presented findings are reported according to the Anatomical Quality Assurance (AQUA) checklist^22^.

### Nerve block technique

Nerve blocks were performed bilaterally, but each side was randomized individually. Thus, totaling eight or nine nerve blocks per injection technique (Table 1). The injectate was a mixture of 38 mL of LA and 2 mL of contrast medium (Visipaque™, GE Healthcare, Eindhoven, The Netherlands) for all techniques. This total volume was chosen as it reflects routine clinical practice, while previous research suggests that this volume may be able to reach the target nerves, including the ON^17,23^. Ultrasound guidance was used to visualize the different anatomical structures, to guide the needle tip to the correct plane, and to observe injectate spread. Nerve blocks were placed by two anesthesiologists specialized in regional anesthesia (WtH, HMB). Study procedures were completed within six hours of arrival at the Amsterdam University Medical Centers.

**Table 1:** A summary of nerve involvement after the different injection techniques.

	S-FICB-H (9 blocks)	S-FICB (8 blocks)	FNB (8 blocks)	I-FICB (9 blocks)
FN, n (%)	9 (100.0)	8 (100.0)	8 (100.0)	2 (22.2)
LFCN, n (%)	9 (100.0)	8 (100.0)	7 (87.5)	4 (44.4)
ON, n (%)	0 (0.0)	1 (12.5)	0 (0.0)	0 (0.0)

*FN* femoral nerve, *LFCN* lateral femoral cutaneous nerve, *ON* obturator nerve, *S-FICB(-H)* suprainguinal fascia iliaca compartment block with or without hydrodissection, *FNB* femoral nerve block, *I-FICB* infrainguinal fascia iliaca compartment block.

S-FICB was applied by puncturing the skin approximately one centimeter cranial to the inguinal ligament in caudal to cranial fashion^9^. In the S-FICB-H group, we subsequently advanced the needle tip an additional five centimeters under the FI (using hydrodissection) in order to achieve a pronounced cranial position not previously documented in the literature for a single-shot technique. I-FICB was performed at the lateral third of the line connecting the pubic tubercle and the anterior superior iliac spine, one centimeter caudal to the inguinal ligament^10^. The injection was placed at the inguinal crease, adjacent to the FN, in case of (ultrasound-guided) FNB.

### Outcome parameters and data analysis

The primary outcome parameter of this study was ON coverage. Additionally, we measured cranial injectate spread as a function of the injectate’s ability to migrate to the debated cranial opening (in the FIC) and ON^12,13^. Further, FN and LFCN coverage were other secondary outcome parameters.

Injectate spread was documented 15 min after injection using whole-body computed tomography (CT) (Siemens SOMATOM Force, Siemens Healthineers, Forchheim, Germany; CT collimation 0.6 mm, dual energy 100/Sn150 kVp, 486/244 mA, pitch 0.6, slice thickness 1.0 mm, soft kernel). A musculoskeletal radiologist blinded to the injection technique assessed the locoregional contrast medium spread. Perineural presence of contrast was assessed by visualization and inspection of the anatomical location of the relevant structures in axial, sagittal and coronal planes, with additional reformatting when deemed necessary. For example, the full path of the ON between its origin and exit through the obturator foramen was inspected. Cranial diffusion was measured in coronal reformatted images from the femoral head (in centimeters) and is also described in relation to the corresponding vertebra.

Data for cranial injectate spread was tested for normality using histograms and the Shapiro–Wilk test, and is presented as mean ± standard deviation. Cranial injectate spread was subsequently compared across the study arms by applying one-way ANOVA and Tukey post-hoc testing. Other outcome measures were summarized as frequencies with percentages. A *P*-value smaller than 0.05 was deemed statistically significant. IBM SPSS Statistics, Version 28.0.1.0, was used for statistical analysis (IBM Corp., Armonk, NY, USA).

## Results

Cadavers were predominantly male (12 of 17, 71%). Further baseline characteristics and details about prior medical history were unavailable, due to the anonymous nature of the body donation program.

### Nerve involvement

**Figure 2:**
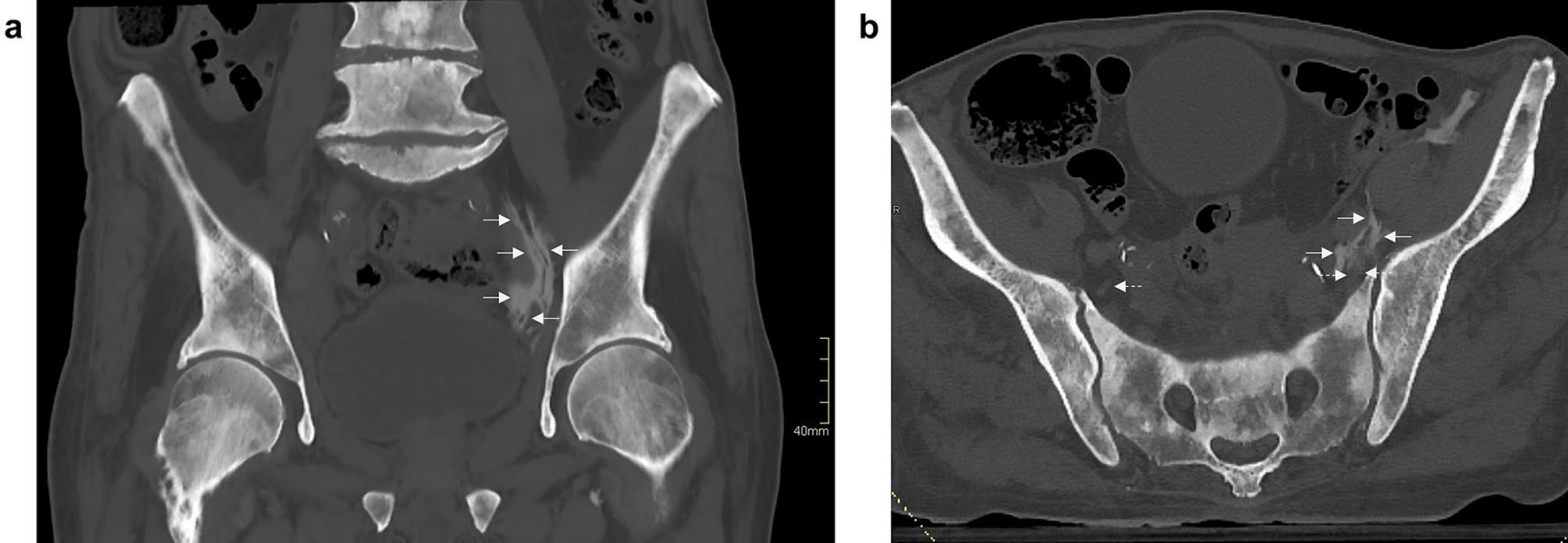
Coronal (**a**) and axial (**b**) CT imagery of the S-FICB case wherein the contrast mixture reached the ON (left side). The injectate was found to involve the ON in the retroperitoneal compartment at the level of the first sacral vertebra, which was also the maximum height that was reached. Dashed arrows point to the ON, while solid arrows point to contrast.

**Figure 3:**
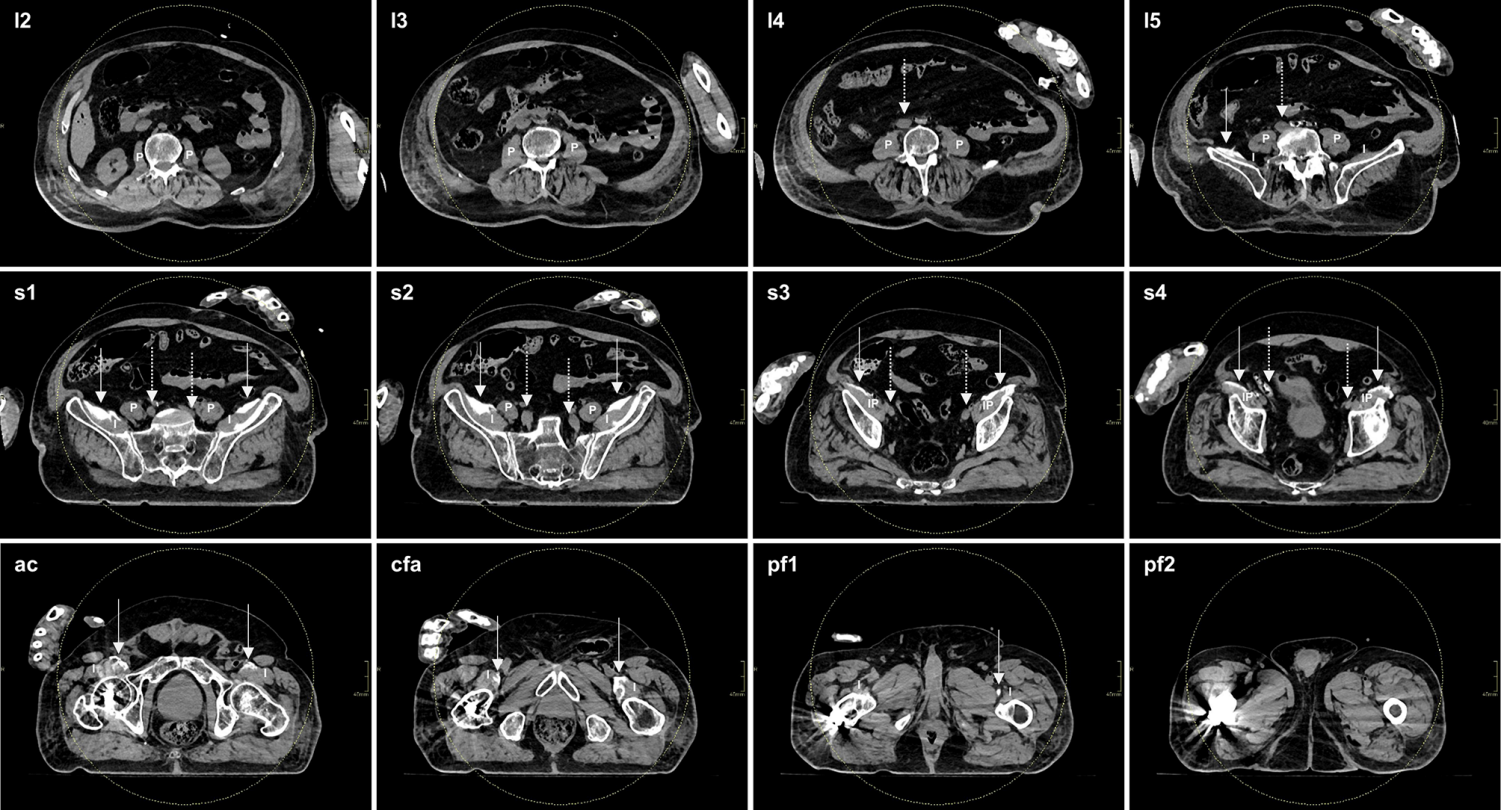
Axial CT slices detailing typical spread of injectate after (bilateral) S-FICB-H. The LA-based contrast mixture was determined to reach a maximum height corresponding to the fifth lumbar vertebra on the left side and the intervertebral disc of lumbar vertebrae four-five on the right side. Medial spread of contrast stayed anterior to the iliopsoas muscles and iliac arteries. As such, contrast remained removed of the location of the lumbar plexus and ON, both in the cranial and medial directions. Dotted arrows point to the iliac arteries, solid arrows point to the contrast. *l* lumbar vertebra, *s* sacral vertebra, *ac* acetabulum, *cfa* common femoral artery, *pf* proximal femur, *P* psoas muscle, *I* iliacus muscle, *IP* iliopsoas muscle.

The ON was involved in one (S-FICB) of 34 nerve blocks, while none reached the lumbar plexus. The nerve block that did reach the ON was found to involve this nerve in the retroperitoneal compartment, at a level corresponding to the first sacral vertebra (Fig. 2). This was also the maximum height reached by the contrast for this block. Medial injectate spread was limited in other cases and did not reach the ON, as exemplified in Fig. 3 after bilateral S-FICB-H.

**Figure 4:**
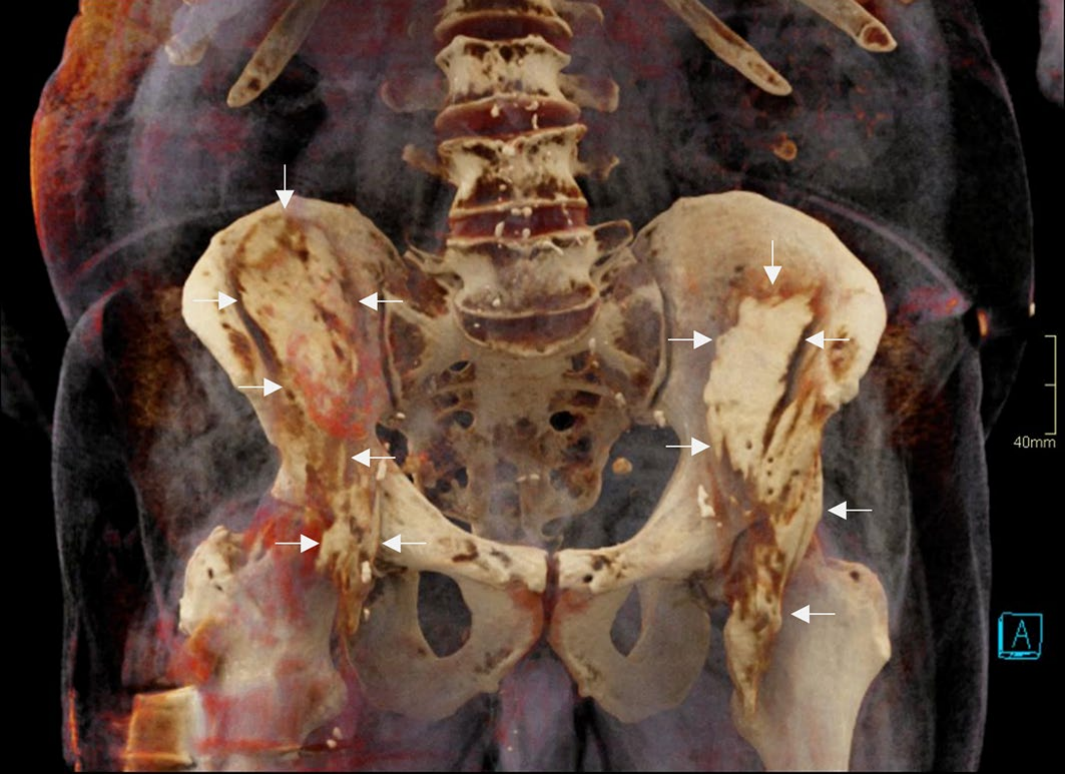
A 3D volume rendered reconstruction of CT-imaging of a specimen that was randomized to receive bilateral S-FICB-H. Contrast reached a maximum height equal to the level of the fifth lumbar vertebra on the left side and the intervertebral disc of lumbar vertebrae four-five on the right side. The arrows point to the contrast.

The FN and LFCN were consistently involved in S-FICB-H, S-FICB and FNB, while these structures were considerably less often involved in I-FICB (22.2% and 44.4% of cases, respectively). Details of nerve involvement are summarized in Table 1. Figure 4 depicts a volume rendering reconstruction of a cadaver with typical injectate distribution patterns after bilateral S-FICB-H.

### Cranial injectate spread

Spread of the injectate towards the lumbar plexus was measured in relation to the femoral head. Truncal spread was found in only two of nine (22%) I-FICBs and as such, I-FICB was excluded from statistical analysis. The mean cranial spread of S-FICB-H (14.00 ± 3.16 cm) was significantly more than that of S-FICB (10.28 ± 2.95 cm, p = 0.046), or FNB (5.30 ± 2.86 cm, p ≤ 0.001). Injectate reached higher for S-FICB than for FNB (p = 0.008). Similar distribution patterns were observed when cranial injectate spread was related to the vertebral column, as shown in Table 2, and demonstrated for S-FICB-H in Fig. 3.

**Table 2:** Cranial injectate spread related to the vertebral column.

	S-FICB-H (9 blocks)	S-FICB (8 blocks)	FNB (8 blocks)	I-FICB (9 blocks)
L2, n (%)	1 (11.1)	–	–	–
…				
L4, n (%)	2 (22.2)	3 (37.5)	–	–
IVD L4-L5, n (%)	1 (11.1)	–	–	–
L5, n (%)	4 (44.5)	1 (12.5)	–	–
IVD L5-S1, n (%)	1 (11.1)	–	1 (12.5)	1 (11.1)
S1, n (%)	–	2 (25.0)	1 (12.5)	1 (11.1)
IVD S1-S2, n (%)	–	2 (25.0)	–	–
S2, n (%)	–	–	3 (37.5)	–
…				
Coccyges, n (%)	–	–	2 (25.0)	–
…				
Below the VC, n (%)	–	–	1 (12.5)	7 (77.8)

*L* lumbar vertebra, *S* sacral vertebra, *IVD* intervertebral disc, *VC* vertebral column, *S-FICB(-H)* suprainguinal fascia iliaca compartment block with or without hydrodissection, *FNB* femoral nerve block, *I-FICB* infrainguinal fascia iliaca compartment block.

## Discussion

This radiological cadaveric study supports the hypothesis that S-FICB-H leads to the most cranial injectate spread of the techniques targeting the FIC. Concurrently, however, our findings also demonstrate that pronounced cranial injectate spread will not result in reliable blockade of the ON. I-FICB resulted in less involvement of the FN and LFCN than S-FICB-H, S-FICB and FNB.

The ON is a mixed sensory and motor nerve that derives from the anterior divisions of the second, third and fourth lumbar spinal nerves. Its branches innervate the adductor muscles of the thigh, and the hip and knee joints. Further, the ON contributes, in some patients, to the sensory innervation of a section of the medial thigh. Therefore, inclusion of ON blockade in FICB would improve analgesia after lower extremity procedures, such as hip fracture surgery.

However, it is still debated whether the ON can indeed be covered during FICB. Clinical examination after FICB may be ambiguous due to the highly variable cutaneous innervation of the ON and co-innervation to the adductor muscles^24^. Meanwhile, (both primary and secondary) cadaveric dissection is prone to disrupt the integrity of fascial layers, and is therefore at risk of false positive research results. This study therefore applied CT-imaging of a LA-based contrast mixture, as it allowed for precise and objective (numerical) measurements of injectate spread. Additionally, CT-imaging allowed us to include 34 samples, a number which would have been unrealistic to obtain with dissection (or magnetic resonance imaging; MRI). Further, the advanced CT scanner used here provided us with similar information as would have been obtained with MRI.

The data presented here for S-FICB (both with and without additional needle advancement) deviates from previously reported success rates of ON involvement, but is closely aligned to reported rates of FN and LFCN blockade^9,11,17^. Literature details differing success rates for I-FICB^8,10,17,25–30^. Interestingly, our results are similar to that of authors who performed I-FICB based on the description that was also the foundation of the technique applied in this study^17,28^. The current study supports the conclusion that S-FICB leads to more cranial spread and better FN and LFCN involvement than I-FICB, yet our results indicate less cranial distribution patterns for conventional S-FICB and I-FICB than previously reported in a volunteer study^17^. The 40 mL injectate allowed FNB to behave as a field block, while the inguinal injection site might have facilitated more efficient migration towards the FN and LFCN than possible for I-FICB.

The ON’s path between its origin and the obturator foramen is of importance as the aforementioned nerve block approaches are all based on the presumption that the ON can be reached by an injectate in the FIC^6,8–11^. However, anatomical reports have shown that the ON does not travel within the FIC and suggested an alternative mechanism of action: cranial diffusion from the FIC to the retroperitoneal compartment^12,13^. Assuming this path exists, lack of ON involvement can be hypothesized to be caused by either an inadequate injection volume and injection force, or a too distal injection site, placing the LA out of reach from the retroperitoneal compartment. This scenario could explain the unusually high—compared to the FIC’s capacity of 23 mL—minimum effective volume of 62.5 mL found in a recent cadaver dissection study that applied a step-up, step-down algorithm to determine the optimal injectate volume^12,14^. However, it is as of yet unknown what effect a large injectate volume, or high injection pressure, has on fascial plane integrity (while concurrently the clinical relevance and utility of a nerve block approach that requires injection of a large number of ampoules can be debated). Similar to needle misplacement in the retroperitoneal compartment, disruption of the fascial compartment could lead to creation of an artificial opening in the FI and diffusion towards the ON^15^. This, however, would not be a *true* fascial plane block and constitutes a different mechanism than sought after with a FICB. We speculate that this mechanism is the explanation for the case in which the ON was successfully involved in our study, and the underlying reason for the limited replicability of studies.

Alternatively, this investigation assessed whether advanced needle insertion into the cranial direction would result in ON involvement. The lack thereof shown here contradicts the existence of an easily accessible cranial route from the FIC to the retroperitoneal compartment. Further, as reported in other studies, posteromedial spread was limited and remained removed from the ON^19,25^. Thus, overall, achieving reliable ON blockade using a FIC approach seems not possible, as illustrated by the presented data, the considerable number of published methodological variations, and the differences in reported success rates between studies that applied the same injection technique.

A limitation of this study is the reliance on (human) cadavers as tissue quality might have deteriorated after death. However, specimens were fresh, unfrozen and unembalmed, allowing for near-normal block procedures as shown by the pattern of spread after nerve blocks.

## Conclusion

In summary, neither S-FICB-H nor any other technique targeting the FIC leads to reliable ON involvement, and as such the ON seems to be unrelated to fascial plane blocks targeting the FIC. Addition of a separate ON block to S-FICB (or large volume FNB) should be considered when clinically indicated.

## Data Availability

The datasets generated and analyzed during the current study are available from the corresponding author on reasonable request.
